# Qualitative simulation of bathymetric changes due to reservoir sedimentation: A Japanese case study

**DOI:** 10.1371/journal.pone.0174931

**Published:** 2017-04-06

**Authors:** Ahmed Bilal, Wenhong Dai, Magnus Larson, Qaid Naamo Beebo, Qiancheng Xie

**Affiliations:** 1 College of Water Conservancy and Hydropower Engineering, Hohai University, Nanjing, China; 2 State Key Laboratory of Hydrology-Water Resources and Hydraulic Engineering, Nanjing China; 3 National Engineering Research Center of Water Resources Efficient Utilization and Engineering Safety, Nanjing, China; 4 Department of Water Resources Engineering, Lund University, Lund, Sweden; Louisiana State University, UNITED STATES

## Abstract

Sediment-dynamics modeling is a useful tool for estimating a dam’s lifespan and its cost–benefit analysis. Collecting real data for sediment-dynamics analysis from conventional field survey methods is both tedious and expensive. Therefore, for most rivers, the historical record of data is either missing or not very detailed. Available data and existing tools have much potential and may be used for qualitative prediction of future bathymetric change trend. This study shows that proxy approaches may be used to increase the spatiotemporal resolution of flow data, and hypothesize the river cross-sections and sediment data. Sediment-dynamics analysis of the reach of the Tenryu River upstream of Sakuma Dam in Japan was performed to predict its future bathymetric changes using a 1D numerical model (HEC-RAS). In this case study, only annually-averaged flow data and the river’s longitudinal bed profile at 5-year intervals were available. Therefore, the other required data, including river cross-section and geometry and sediment inflow grain sizes, had to be hypothesized or assimilated indirectly. The model yielded a good qualitative agreement, with an R^2^ (coefficient of determination) of 0.8 for the observed and simulated bed profiles. A predictive simulation demonstrated that the useful life of the dam would end after the year 2035 (±5 years), which is in conformity with initial detailed estimates. The study indicates that a sediment-dynamic analysis can be performed even with a limited amount of data. However, such studies may only assess the qualitative trends of sediment dynamics.

## Introduction

Sediment transport modeling is a process of using a numerical or physical model to reproduce the transport processes of a real river system in a controlled environment. Mathematical modeling provides useful knowledge about a river and dams[1] built on it and can able to predict the effect of different external factors on bathymetric changes and reservoir sedimentation. This knowledge assists efficient and sustainable development related to rivers [2]. Reservoir sedimentation not only negatively affects the storage capacity of a reservoir [3], but it also alters the current biogeochemical and ecological cycles[4–7]. Sediment-dynamics analysis provides information about bathymetric changes upstream of a reservoir. A numerical model may be used for this analysis to predict the upcoming trends of bathymetric changes under different scenarios. This process is a complex task that requires a significant amount of observed data[8]. Among other factors, the prediction quality of a numerical model also depends on the spatiotemporal resolution of the observed records, which include flow time series, sediment inflow and outflow time series, river cross sections, and river bed soil data [9].

All the required information is not always available in the desired quality; therefore, a modeler must make logical assumptions or assimilate data indirectly. The purpose of this study is to demonstrate that by utilizing existing knowledge and available tools it is possible to predict trends in bathymetric changes upstream of a reservoir qualitatively.

Hobgen, Myers [10] worked with these restrictions to create a sediment budget for a small catchment in Indonesia; specifically, they used freely available satellite imagery, interviews with residents, and radionuclide tracers as tools to overcome a lack of observed records. Maswood and Hossain [11] developed a flood forecasting one-dimensional (1-D) model for the Ganges–Brahmaputra–Meghna (GBM) basin. In their case, valuable observed data was available only for 7% of the basin area, so they also used proxy approaches to constructing the remaining data by using satellite imagery and climate data. In another study, Md Ali, Solomatine [12] used different qualities of topographic data in 1-D hydraulic modeling. All these studies show that the unavailability of observed data can be overcome by using alternative approaches and valid hypotheses.

This study is different from the studies above in two ways. First, this study focuses on sediment dynamics by modeling bathymetric changes upstream of a dam when no cross section data is available and only annually averaged flows are available, which, according to authors' knowledge, has not been done before. Second, the present study mainly uses proxy approaches to overcome low spatiotemporal resolution of flow data and a simplified approach to overcome channel cross-section availability.

This study selects a 32 km reach of the Tenryu River just upstream of Sakuma Dam in Japan. Hydraulic Engineering Center's River Analysis System (HEC-RAS), which is widely used for 1-D river analysis, is used as the modeling tool. The primary objective of this work is to analyze the predictive capability available data and existing tools when some of the relevant data (i.e., channel cross sections, riverbed grain size distribution, and monthly river flow) is obtained through assumptions and derived from different sources of varying quality.

## Case study

### Tenryu River and Sakuma Dam

**Fig 1:**
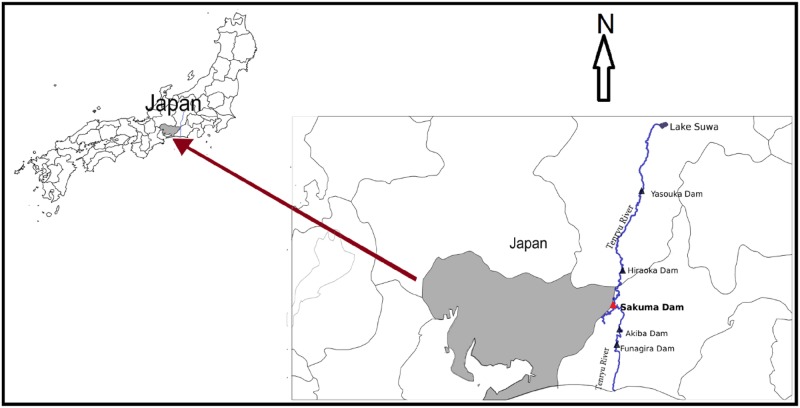
Tenryu River and its dams, the selected reach of Tenryu River is also identified.

The Tenryu River is among the major rivers of Japan, with a total main stream channel length of 213 km and a catchment area of 5090 km^2^ [13]. The catchment is located where the median tectonic line passes [14]. It originates from Lake Suwa, flows toward the south of Japan, and ultimately falls into the Pacific Ocean at Enshu coast (Fig 1). The delta of the Tenryu River is formed to the west of Shizuoka Prefecture. The Tenryu River has good hydroelectric potential, i.e., 350 MW, owing to its high volume of flow and relatively steep slopes. Most of its flow path is mountainous, where it passes through a valley shaped path. The annually averaged discharge is 112 m^3^/s based on records of river flow during 1939–1992 [13]. Because of the sloped terrain and frail geologic composition of the catchment, the sediment discharge of the Tenryu River is considered to be one of Japan's highest, being approximately 3.8×10^7^ m^3^/yr [14]. There are five major dams along the main river, namely, Yasuoka Dam, Hiraoka Dam, Sakuma Dam, Akiba Dam, and Funagira Dam (Fig 1). The largest one is Sakuma Dam [15], which was the 10th biggest dam in Japan at the time of its construction in 1956.

Sakuma Dam, which became operational in 1957, is located in Toyone city of the Kitashitara District on the border of Aichi Prefecture. The length and height of its embankments are 293.5 m and 155.5 m, respectively [16]. It is a concrete gravity dam located in the mountainous area where the Tenryu River flows through a valley shaped terrain. Sakuma Dam is located approximately 70 km upstream of the river mouth. The upstream dam on the Tenryu River is Hiraoka Dam. The total reach of the Tenryu River between Hiraoka Dam and Sakuma Dam is approximately 32 km.

**Fig 2:**
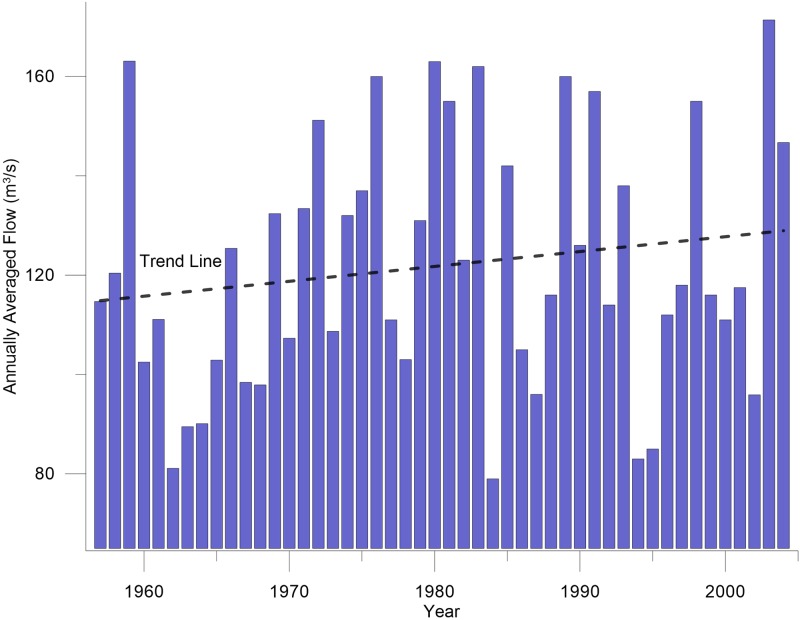
Annually averaged flow history for Sakuma Dam (1957–2004).

Normally dams have a well-developed lake near its embankment and water levels in the river upstream of the lake are not affected by the storage conditions in the reservoir. However, because Sakuma Dam’s reservoir lies in a hilly area, its banks are steep, so it does not have a well-developed lake like normal dams. Therefore, it stores water along the length of the river, and the effect of storage sometimes reaches even just downstream of Hiraoka Dam. The annually averaged flow typically varies from 79 m^3^/s to 171 m^3^/s (Fig 2).

The dam was constructed to exploit the hydropower generation capacity of the Tenryu River. However, since 1957, when it started operating, it has continuously faced the problem of sediment retention, which has resulted in the reduction of storage capacity. An average sediment load of 2×10^6^ m^3^ is retained each year[14]. This huge amount of sediment retention is not only jeopardizing the dam's useful life, but it is also creating problems downstream of the dam. The water that leaves Sakuma Dam has less coarse sediments; therefore it can erode the riverbed downstream to balance its energy. The riverbed at the downstream end has been degraded by 1 m to 1.5 m [14]. The sedimentation in upstream reservoirs has also caused coastal erosion.

### 1.1 Data availability

Available observed data for 1956–2004 includes (1) river bed profile at five-year intervals, (2) incoming and outgoing sediment load, and (3) records of a few peak flow events along with respective sediment data during those events.

## Construction of the numerical model with HEC-RAS

### Data input and boundary conditions

For sediment-dynamics modeling in the reservoir, the required input data can be categorized into three types: (1) geometric data, (2) quasi-unsteady flow data, and (3) sediment data.

#### Geometrical data

Geometric data in HEC-RAS consists of linking a particular number of river cross sections along the full reach to create the schematic river system. The modeled portion of the Tenryu River was a 32-km reach from Hiraoka Dam to Sakuma Dam that was divided into 48 river stations. The data necessarily included stations and elevations for each cross section. Additionally, Manning's *n* for left overbank (LOB) channel, and right overbank (ROB), as well as contraction and expansion coefficients were required to create the geometric data file. LOB and ROB are regions of channel cross-section out of the main channel and are on left and right sides accordingly. Coefficients of contractions and expansion account for the energy losses in a channel due to contraction and expansion.

Google Earth was utilized to acquire the detailed geometric data to be used in the model, i.e., the river width and downstream lengths at different cross sections along the reach. Then, AutoCAD was used to draw the vectorized river schematic to scale. A trapezoidal geometric shape was assumed for all cross sections.

#### Flow data

The sediment transport simulations are dependent on the quasi-unsteady hydraulics data. In this study, sediment load carried into Sakuma reservoir was required from 1957 to 2004. Annually averaged flows were available, and these flows were converted into monthly averages for all years (i.e., 1957–2004) based on precipitation measurements in the catchment. This method was based on the hypothesis that, as the catchment of the Tenryu River is a hilly area, the response time of rainfall would be very quick compared to the time scale of the flow model. This hypothesis is also supported by some other studies [17, 18], which describe that an antecedent rainfall in the watershed is one of most relevant factors controlling the hydrological response of a river. Based on this hypothesis a simple relationship (Eq 1) was developed between precipitation and runoff to convert annually averaged flows to monthly averaged flows:

$$Q_{m.avg}=\frac{12P_{m}Q_{y.avg}}{P_{y}},$$ (1)

Where

Q_m.avg_ = monthly average flow (m^3^/sec),

q_y.avg_ = yearly average flow (m^3^/sec),

p_m_ = monthly precipitation (mm),

p_y_ = yearly precipitation (mm),

m denotes month [m = 1, 2, …, 12], and

y denotes year [y = 1957, 1958, …, 2004].

A flow history was used as the boundary condition in the most upstream river station. This boundary condition consists of the amount of flow, duration, and computational increment. The computational increment was initially set to be ten days in simulations.

For the downstream boundary condition, a stage–discharge rating curve was developed to model the effect of the dam embankment on the discharge. The discharge structures of the dam were described in a simplified manner to yield the appropriate flows at the minimum and maximum water levels in the dam. Thus, it was assumed that flow through the dam was only through a submerged gate and follows equations rules of flow through a submerged orifice.

$$Q=Q_{max}\;{\left(\frac{z-z_{n}}{z_{max}-z_{n}}\right)}^{2},$$ (2)

Where:

Q = river inflow at any instant of time *t* in the river reach to be modeled (m^3^/sec)

q_max_ = maximum monthly flow that may happen during simulation period (m^3^/sec)

z = water level in the dam at time *t* (i.e., stage)

z_max_ = maximum water level in the dam (260 m)

z_n_ = water level at the outlet (180 m)

**Fig 3:**
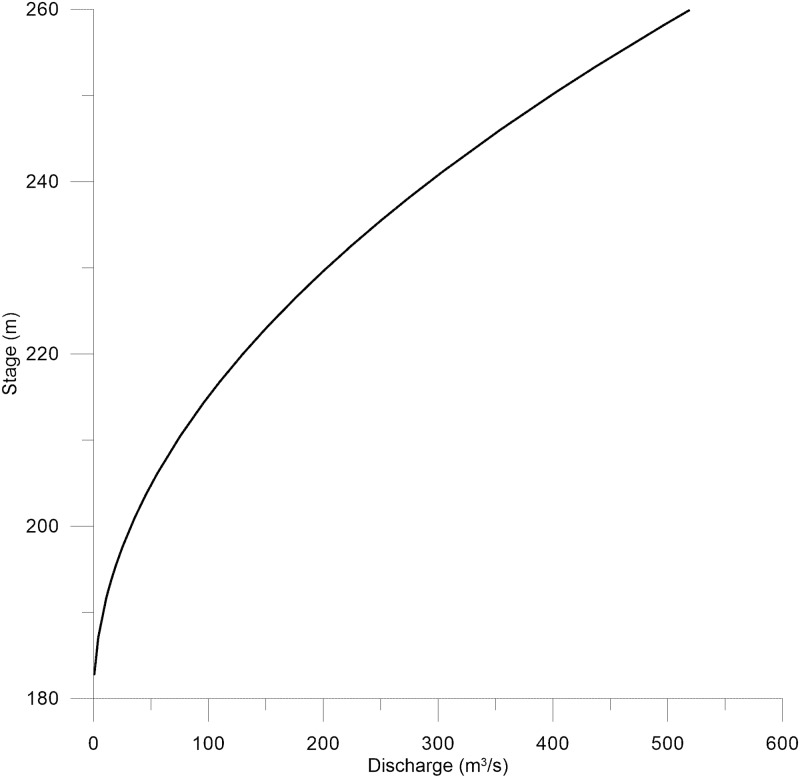
The stage–discharge curve used as a boundary condition in quasi-unsteady flow data in HEC-RAS.

The stage–discharge curve obtained through the equation is shown in Fig 3.

#### Sediment data

The sediment data consists of the following main input quantities.

i Bed gradation at each river station

**Fig 4:**
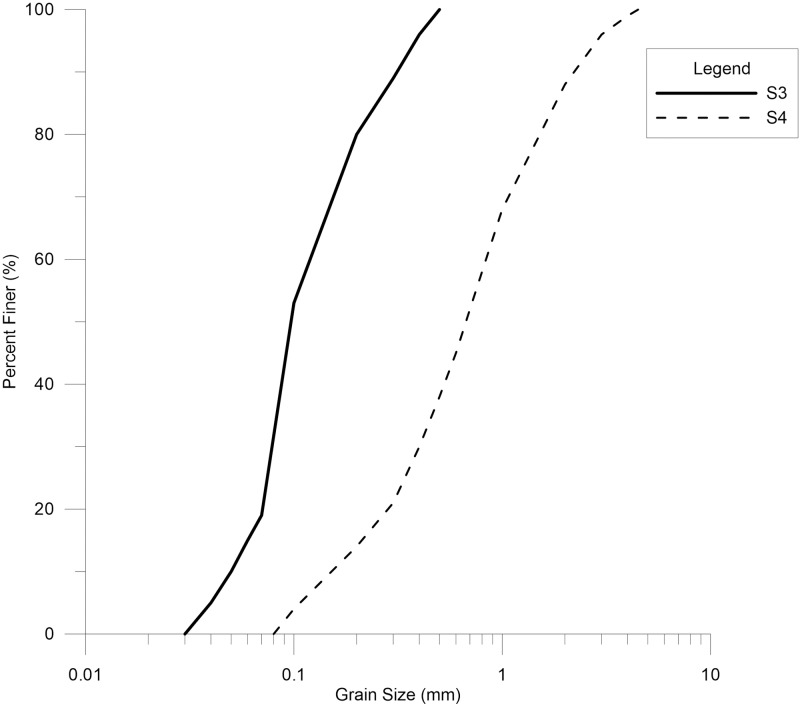
Grain size distribution for river bed of soil types S3 and S4 used in the model as initial conditions for 1957. Data were taken from Morris and Fan [19].

The soil gradation of the Tenryu River bed in 1957 was unknown; however, Morris and Fan [19] presented a gradation curve for the river, which was utilized in this study. Although the figure shows bed gradation curves for the river bed in 1982, the same profile was adopted for 1957 as well. However, during calibration, better results were obtained when only S3 and S4 were used for the full reach (Fig 4).

ii General sediment properties

The following values were used for modeling

Specific gravity = 2.65,

Shape factor = 0.6,

Density of sand/gravel = 1489 kg/m^3^,

Density of silt = 1041 kg/m^3^, and

Density of clay = 480 kg/m^3^.

i Sediment boundary conditions

The boundary condition for the upstream river station was initially the "equilibrium load" and a sediment "rating curve" was the downstream boundary condition. The sediment rating curve provided the amount of sediment load that was exiting at the most downstream river station throughout the simulation. Therefore, the model makes use of the rating curve to calculate the outgoing sediment load from the most downstream river station, makes a bed change calculation based upon the transport function, sediment properties, and other hydraulic parameters, and gradually reaches most upstream river station. At the upstream river station, the "equilibrium Load" boundary condition was selected, which means that the sediment coming into the system is based on its transport capacity. The transport capacity is determined by prevailing hydraulic and sediment parameters [20].

The sediment rating curve was developed from available data using the hypothesis that a regime-type equation (Equation 4) governs sediment influx. The regime-type equations are empirical relationship between sediment load associated with a particular stream flow. Such equations give a good estimate of sediment load for a particular flow condition in the absence of any field measurement and have been used in many similar studies e.g. by Mohammad, Al-Ansari [1], Zeleke, Moussa [21] and Gomez, Cui [22]. Thus, from known values of sediment inflow against river flow, a regime type equation was developed:

$$q_{s}=0.063Q^{1.7},$$ (3)

Where

q_s_ = Sediment load (ton/day), and

Q = River flow (m^3^/s).

### Assumptions and limitations

During modeling, not all the data were readily available, and assumptions and simplifications had to be made in several parts of this study. These assumptions included:

1. A trapezoidal channel cross-section shape was assumed instead of the original cross-sections. However, it was made consistent with the average depth and top width of the river.
2. It was assumed that the bed gradation of the river is constant throughout the river reach at the start of the model. Additionally, the actual bed gradation was not available before Sakuma Dam was constructed; however, the bed gradation from 1982 at four places was known. However, two of these curves—bed gradation curves 3, and 4 (Fig 4)—were used as the initial bed gradation.
3. The fraction of different grain sizes in the incoming sediment load was not available, so the bed gradations from 1982 were used to obtain this input. These sediment grain sizes were used as the initial sediment input and then changed during the calibration processes.
4. Only the annually-averaged flow data from Sakuma Dam was available. As this data was not at the desired temporal resolution for modeling, it was assumed that rainfall response time is small compared to the flow modeling time step because the river reach is in a hilly area. Using this assumption the annually averaged flow data was extrapolated to monthly flow data based on the measured monthly precipitation in the catchment.
5. The flow rating curve that was used as the downstream boundary condition in the quasi-unsteady flow data was developed based on general information about the discharge and the water level in the dam.

There are two types of limitations with this modeling: those that are inherent to the HEC-RAS model and those that are the result of a lack of input data. These restrictions make it necessary to make different assumptions and simplifications:

1. The model is based on a 1-D algorithm, which means that it can only model the sediment transport along the length of the river and it does not take into account velocity variations along the depth or width of the river.
2. Japan is in a zone of high seismic activity [23], which might have some effects, in particular on the shape of the delta by the energies released by earthquakes. For example, Khan, Gul [24] observed a change in topset slope—due to seismic activities—of Tarbela Dam in Pakistan. However, HEC-RAS does not model such effects on the bathymetry.
3. Transverse movement of water, meandering, and point bar formation were not modeled.

## Results

HEC-RAS is designed to perform 1-D steady and unsteady flow, sediment transport, and water quality modeling. Modeling sediment transport behavior is not an easy task, as in addition to theoretical derivations it also has some form of empiricism, i.e. based on experimental data and is not derived from laws of physics. Further, it also contains a broad range of very sensitive physical parameters [25]. HEC-RAS uses a hydrologic simplification called the quasi-unsteady flow method, which approximates a continuous hydrograph with a series of discrete steady flow profiles [25].

HEC-RAS performs sediment routing by solving a simplified 1D formulation that the rate of change in the bed elevation over time, in a control volume is proportional to the difference between sediment inflow and outflow. It computes the sediment transport capacity in a control volume and then compares it with sediment inflow. If the inflow exceeds the transport capacity, aggradation will occur; however, if the transport capacity exceeds the inflow, degradation will occur.

### Calibration

Sediment aggradation or degradation in the river upstream of Sakuma Dam was represented by the change in the channel invert over time. As previously discussed, the channel’s cross-sectional shapes were not available; therefore, for simplification, a trapezoidal cross section was assumed. Furthermore, since this cross section may differ from the actual one, it is hard to compare and relate the model result regarding sediment volume. This barrier is caused by the difference from actual initial cross-sectional geometries in the model, implying that different cross-sectional areas for the same amount of sediment deposition or erosion would have produced different bed profiles. Therefore, the channel invert was selected as the criterion for comparison of model results with observed values during the calibration and validation of the model.

**Fig 5:**
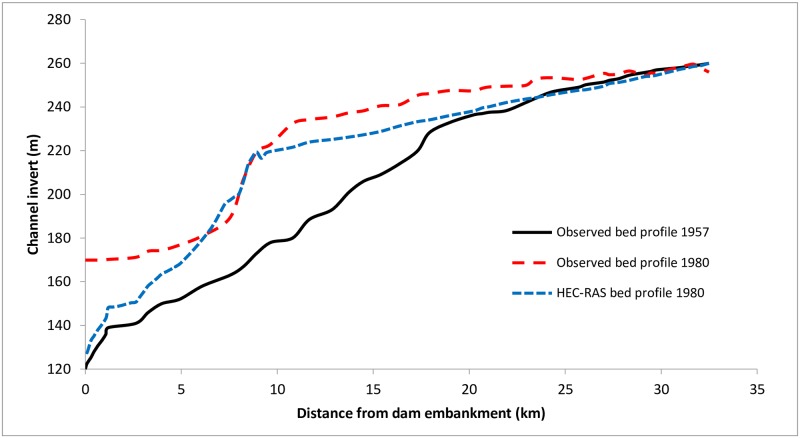
Comparison between observed and HEC-RAS bed profile of 1980 after calibration.

Initial sensitivity analysis of the model suggested that the model results are quite sensitive to bed gradation, which was likewise observed in the calibration process. The bed profile selected for model calibration at the start of simulations was a variant of the bed gradation at different locations from the bed profiles of 1982. The observed channel invert in 1980 was used for the calibration of the model. The coefficient of determination, R^2^, was 0.79 for the bed profile obtained from calibration. Because many input data were assumed or indirectly extracted, this R^2^ value was considered satisfactory, and the model was taken as satisfactorily calibrated (Fig 5).

Hiraoka Dam is located just before the most upstream river station used in the model. This dam is already filled with sediments, so the entire incoming sediment load is consequently transported through the dam without deposition. For this reason, there is very less degradation taking place at the river stations close to Hiraoka Dam, neither in the observed bed profile nor the modeled bed profile.

The most important parameter values of the finalized calibration included:

1. Manning's *n* was a mix of 0.03, 0.035 and 0.04 for different river stations along the full reach.
2. Toffaletti's [26]method was used for the transport function, Exner 5 [20, 25, 27]was utilized for the sorting method is, and the fall velocity was calculated using the Van Rijn equation[28].
3. The sediment rating curve was calculated using (Eq 3)
4. The initial bed gradation is a mix of the two bed gradation samples (Fig 4). Soil type S3 consists of relatively fine-grained particles; therefore, S3 was assigned for the downstream half of the simulated river reach. In contrast, S4 was assigned for the upstream half of the river reach.

These final parameter values are rational for the following reasons:

- Toffaletti's method is considered a sediment transport function for "large river" systems [25], and the model is used to simulate Sakuma Dam and the Tenryu River, which is among the largest rivers of Japan [13, 29]. The grain-size validity range for Toffaletti's method is from 0.062 mm to 4 mm [25], and the final grain sizes used for calibration—and later for validation—were in the same range (Fig 4). For soil type S4 (Fig 4) approximately 7% of the soil had a grain size between 4 mm and 8 mm, which is slightly above the validity range of Toffaletti's method; however, this small deviation can be neglected.
- Applicable conditions of the Van Rijn fall velocity method are also in line with the actual environment of the Tenryu River. The Van Rijn Method calculates particle fall velocity using a shape factor of 0.7 [25], which is the same as that of natural sand [30]. It can be seen from the grain-size distribution curves (Fig 4) that most of the bed material consists of sand.

### Validation

After calibration had been performed for the 1980 bed profile, the model was validated for the intermediate bed profiles of 1975, 1989, 1993, 1998, 2003, and 2004. Unfortunately, compared to the results for the 1980 bed profile during the calibration process, the calibrated model did not yield quite as good agreement for some cases.

**Fig 6:**
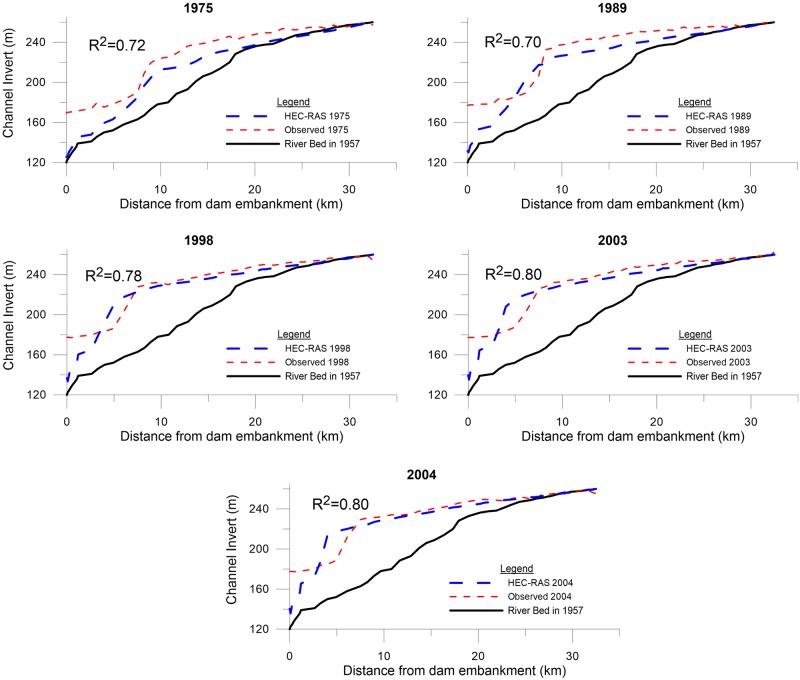
Comparisons of observed and HEC-RAS bed profiles for years 1975, 1989, 1998, 2003, and 2004.

However, the validation results matched the measurements within satisfactory limits. Fig 6 shows the model-generated river bed profiles for selected years and their comparison with the observed river bed profiles.

In addition to the visual resemblance between the model-generated bed profiles and the observed data, the deviation between the model results and observed data were quantitatively estimated. These measurements included calculating R^2^, the percentage difference of area under channel invert, and dam fill-up ratio.

### Predictive scenario

After the calibration and validation the model to a satisfactory level, it was used to forecast possible future bathymetric changes using different future flow conditions. Thus, initially, the model was utilized to simulate bathymetric changes until 2040 for with reusing the same flow history, i.e., 1957–2004. Simulation with repetition of the flow history after 2004 has its importance because it involves natural variation in flow occurrences. This change reflects the prospective behavior of sediment transport, providing the most realistic flow conditions.

A trend line was fitted to the flow history to approximate the trend of flow variation, (Fig 2) from 1957 to 2004. However, the trend line shows only a slightly increasing tendency. Therefore, the flow history from 1957 to 2004 was assumed for the flow "history" after 2004. Thus, the predictive modeling was performed by repeating the flow history. In this scenario, the model parameters and river flow until 2004 remained the same as those used for the calibration. However, the flow values from the observed flow history were used from 2005 to 2040 to forecast the river behavior during these years.

Model results were compared with observed data using the following approaches:

1. Visual resemblance between the modeled and observed bed profiles
2. R^2^ (coefficient of determination) between the modeled and observed bed profiles
3. Observed versus modeled dam fill-up ratio, i.e., loss of accumulative storage due to sedimentation

### Visual resemblance

At the time of model development and comprehensive testing, several input data were either assumed or indirectly assimilated. Initially, the main input data available were annually-averaged flow, channel invert at the start of the simulation and selected years after that, and approximate top width. Therefore, initially, the quantitative comparison of observed sediment volumes was not given high priority. Instead, the visual resemblance between the modeled and observed bed profiles was considered to check the results of a specific model run quickly.

**Fig 7:**
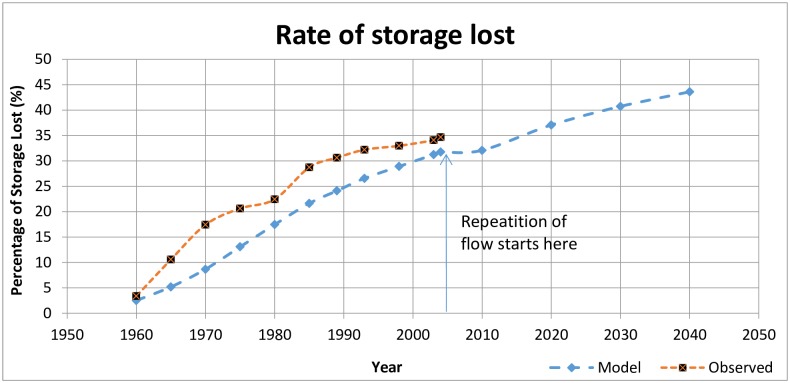
Comparison of rate of storage lost between modeled and observed reservoirs. The line showing modeled values also shows predictive loss of storage after 2004.

During calibration and validation (Figs 6 and 7, respectively), the slope of the modeled bed was nearly consistent with the observed slope, but the modeled channel bed level was slightly lower than the observed level. The location of the delta is a bit ahead of the observed location, causing a slight overestimation of the behavior in this region. Near the embankment, the modeled bed profile again drops below the observed profile—in this region, the difference is relatively high.

In general, the underestimation at the start and end of the bed profile may be attributed to the low temporal resolution of the input flow data—specifically, because the representation of extreme events was not satisfactory. Because a significant amount of sedimentation may occur during high flood events, the lack of modeling such events may be the primary reason for underestimating the bed evolution in the model. Even the largest flows achieved through this process were smoothed over a time frame significantly longer than that of an extreme event.

The reason for the delta location being closer to the dam embankment in the model than that of the observed bed profile could be due to dredging measures at the site, which were not modeled. Additionally, there is high sedimentation close to the dam embankment in observed profiles as compared to model profiles (Fig 6). In reality, some sediment removal measures were undertaken in Sakuma Dam that may also affect and cause minor destabilization in the delta owing to its steeper slope. These events may have caused small soil mass sliding from the delta region to the area near the embankment. Another possible reason of land sliding could be the high seismic activity in Japan, as it is an earthquake-prone zone that has almost 400 minor earthquakes daily [23]. It is beyond the capability of HEC-RAS to model the changes in river bed due to earthquakes or any other external forces.

### Coefficient of determination—R^2^

Quantitatively, the model results can be compared to the observed data by calculating the coefficient of determination, R^2^. It is a statistical measure that estimates how well the model-generated results match the observed data. Shown in Table 1, the R^2^-values for of all available profiles vary from 0.68 to 0.96, which indicates good agreement between the model results and the observed data for most of the profiles.

**Table 1:** Calculated R^2^ for selected years.

S No.	Year of comparison	R^2^ value
1	1960	0.96
2	1965	0.79
3	1970	0.71
4	1975 ^1^	0.72
5	1980 ^2^	0.79
6	1985	0.68
7	1989 ^1^	0.7
8	1993	0.72
9	1998 ^1^	0.78
10	2003 ^1^	0.8
11	2004 ^1^	0.8

^**1**^ Fig 6

^**2**^ Fig 5

### Loss of storage

The loss of storage capacity in the simulated river was calculated as a percentage of initial reservoir volume and compared with the observed loss. These percentages (Table 2) also confirm the underestimation of sedimentation in the reservoir by the model.

**Table 2:** Comparison of storage lost storage due to sedimentation.

Year	Percentage of cumulative storage lost due to sediment accumulation (%)	
	Model	Actual
1960	2.52	3.39
1965	5.19	10.58
1970	8.67	17.44
1975	13.08	20.63
1980	17.46	22.44
1985	21.64	28.73
1989	24.11	30.61
1993	26.57	32.20
1998	28.91	32.97
2003	31.25	34.08
2004	31.73	34.68
2010	32.08	
2020	37.07	
2030	40.73	
2040	43.60	

Morris and Fan [19] discussed that interference with the original design purposes of a dam occurs at a significant level when half of the volume of the dam is lost due to sedimentation, and sometimes even earlier. Thus, it may be assumed that Sakuma Dam’s useful life would end when 50% of its storage volume is lost. Considering the general underestimation (Fig 7) and uncertainty in the model simulation results (Table 2), the useful life of Sakuma Dam can be reasonably expected to end in 2035 (±5 years) if no measures are taken to limit sedimentation.

## Discussion

In general, the model performed satisfactorily once all parameters were set within their effective ranges. Despite many simplifications, HEC-RAS has produced reasonable results with the data available for the period of study. The model results have R^2^-values ranging from 0.68 to 0.96, and the relative difference in river bed profile change between the modeled and observed data is in the range 8%–75%. The relative difference is greater at the beginning of the simulation period when the changes are smaller, but it decreases toward the end of the simulation period. The relative loss of storage volume due to sedimentation is also similar in the observations and the model results.

The river bed consists of sand, which is within the range of applicability of Toffaletti’s transport function, and this gave the best results as compared to other available options. However, the Englund–Hansen formula also gave somewhat reasonable results for some of the test runs. The Van Rijn equation, which was used to predict the fall velocity, produced the best agreement during the calibration phase.

The same model was simulated through 2040 to forecast bathymetric changes. Flow data was not available after 2004; therefore, it was assumed that the flow is periodic as from 1957 to 2004. Model slightly over predicts the storage lost, but since we are only in qualitative trend it may be acceptable. Here it is worth noting that similar over prediction were also reported by Zeleke, Moussa [21] when they used SRH-1D model for prediction of sediment transport in Angereb Dam reservoir. This predictive study shows that after 2035 (±5 years), Sakuma Dam will no longer be useable for its intended purposes without intervention to reduce sedimentation. It is therefore highly recommended that some cost-effective de-sedimentation measures be taken well before this time. One or more of the following possible solutions for sediment management may be applied:

- Watershed management of the Tenryu River [31, 32] may be an option for reducing sediment inflow into the river from its basin by regulating land-use types.
- Dredging of deposited sediments can be performed, especially in the delta area, to modify the shape of the delta and to reduce sedimentation [31] in dead storage capacity. This method, however, cannot be used to remove approximately 5%–10% of sediments owing to its cost and arrangements.
- Sediment bypassing, which has been effectively utilized for some other dams in Japan like Nunobiki Dam and Asahi Dam [33], may be an option. A system of tunnels may be introduced at the bottom of the dam embankment to allow incoming sediment depart with a much-reduced deposition in the upstream region. However, judging the effectiveness of any method used requires a detailed feasibility analysis.

## Conclusion

Conventional methods for gathering data are costly, and historical flow and sediment data are not always available. Even if the data are available, it may not be in the desired spatiotemporal resolution, particularly for transboundary river basins in developing countries. This study explored the possibility of developing river hydraulic models using simplifications, hypotheses, and freely available data and tools (e.g., Google Earth) to overcome a lack of observed data.

With this aim, the focus of this study was to simulate bathymetric changes taking place in the Tenryu River due to the construction of Sakuma Dam. The simulations were further used for prediction of possible future changes in river bed profile and the estimation of when Sakuma Dam will not be able to provide all its intended purposes.

Since the model simulated the qualitative trends satisfactorily, i.e., R^2^ from 0.68 to 0.96, such simulations may be performed for other similar cases with suitable hypotheses and proxy methods. The following aspects are highlighted about the 1-D modeling of the river system to simulate qualitatively the bathymetric changes:

1. Exact cross-sections of a river may not be required, and an assumed cross-section that does not deviate from the actual average depth and top river width can also be used.
2. Low temporal resolution of flow data may be correlated precipitation events to improve temporal resolution. However this hypothesis is probably valid more for watersheds characterized by steep or hilly terrain.
3. River bed soil gradation and Manning’s *n* are the two most important parameters for numerical simulation for bathymetric studies. Therefore, it is critical to use these two parameters as per actual conditions.

Here this is to be emphasized that a qualitative simulation like this not intended to replace the conventional modeling exercise aided with detailed field measurements of observed data. Hence it must not be used for decision making. Instead these studies only aid to conceptually understand the future trends in bathymetric changes and aid for planning of further detailed study

## Supporting information

S1 Table Averaged annually flow. (DOCX)

S2 Table Averaged monthly flow (extracted). (DOCX)

S3 Table HEC–RAS geometry data (x-coordinates, elevations and lengths). (DOCX)

S4 Table Precipitation data in the catchment of Tenryu River. (DOCX)

S5 Table Temperature in the region around Sakuma Dam (°C). (DOCX)
