# Supplementary material for: Qualitative simulation of bathymetric changes due to reservoir sedimentation: A Japanese case study
Source: PLoS One. 2017 Apr 6;12(4):e0174931. doi: 10.1371/journal.pone.0174931 (PMC5383045; doi:10.1371/journal.pone.0174931)
Supplement: S1 Table — (DOCX) [file pone.0174931.s001.docx]

# Averaged yearly flow

Table S1: Actual annually averaged flow (m^3^/s) (Taken from Civil Engineering Department, University of Tokyo, Japan)

| **Year** | **Flow** | **Year** | **Flow** | **Year** | **Flow** |
| --- | --- | --- | --- | --- | --- |
| **1957** | 114.7 | **1973** | 108.7 | **1989** | 160 |
| **1958** | 120.4 | **1974** | 132 | **1990** | 126 |
| **1959** | 163.1 | **1975** | 137 | **1991** | 157 |
| **1960** | 102.5 | **1976** | 160 | **1992** | 114 |
| **1961** | 111.1 | **1977** | 111 | **1993** | 138 |
| **1962** | 81.1 | **1978** | 103 | **1994** | 83 |
| **1963** | 89.5 | **1979** | 131 | **1995** | 85 |
| **1964** | 90.1 | **1980** | 163 | **1996** | 112 |
| **1965** | 102.9 | **1981** | 155 | **1997** | 118 |
| **1966** | 125.4 | **1982** | 123 | **1998** | 155 |
| **1967** | 98.4 | **1983** | 162 | **1999** | 116 |
| **1968** | 97.9 | **1984** | 79 | **2000** | 111 |
| **1969** | 132.4 | **1985** | 142 | **2001** | 117.5 |
| **1970** | 107.3 | **1986** | 105 | **2002** | 95.9 |
| **1971** | 133.4 | **1987** | 96 | **2003** | 171.4 |
| **1972** | 151.2 | **1988** | 116 | **2004** | 146.7 |
