# Supplementary material for: Qualitative simulation of bathymetric changes due to reservoir sedimentation: A Japanese case study
Source: PLoS One. 2017 Apr 6;12(4):e0174931. doi: 10.1371/journal.pone.0174931 (PMC5383045; doi:10.1371/journal.pone.0174931)
Supplement: S2 Table — (DOCX) [file pone.0174931.s002.docx]

# Averaged monthly flow (extracted)

Table S2: Extracted monthly flow based upon precipitation (m^3^/s)

| **Year** | **Jan.** | **Feb.** | **Mar.** | **Apr.** | **May** | **Jun.** | **Jul.** | **Aug.** | **Sep.** | **Oct.** | **Nov.** | **Dec.** |
| --- | --- | --- | --- | --- | --- | --- | --- | --- | --- | --- | --- | --- |
| **1957** | 26.40 | 62.91 | 34.31 | 98.86 | 154.82 | 209.36 | 141.82 | 223.15 | 231.58 | 49.85 | 81.79 | 61.55 |
| **1958** | 82.76 | 99.98 | 68.48 | 104.58 | 79.74 | 41.45 | 241.75 | 149.25 | 262.27 | 155.08 | 65.46 | 94.01 |
| **1959** | 95.81 | 189.96 | 100.20 | 270.02 | 130.17 | 92.19 | 140.48 | 402.00 | 193.16 | 112.61 | 102.78 | 127.80 |
| **1960** | 22.48 | 10.97 | 91.37 | 150.86 | 141.96 | 131.90 | 160.07 | 225.27 | 100.94 | 68.28 | 87.67 | 38.23 |
| **1961** | 49.74 | 21.75 | 113.98 | 225.36 | 168.31 | 290.17 | 46.00 | 111.72 | 55.24 | 130.53 | 92.23 | 28.16 |
| **1962** | 11.43 | 6.61 | 32.05 | 120.42 | 128.88 | 165.15 | 133.65 | 122.73 | 57.45 | 62.34 | 83.91 | 48.57 |
| **1963** | 2.84 | 19.49 | 62.64 | 126.47 | 245.96 | 179.39 | 110.95 | 107.46 | 69.62 | 97.43 | 30.70 | 21.04 |
| **1964** | 104.18 | 54.85 | 73.03 | 105.06 | 56.43 | 299.12 | 28.92 | 59.54 | 187.54 | 62.53 | 21.29 | 28.69 |
| **1965** | 26.61 | 22.97 | 30.61 | 107.29 | 265.51 | 154.71 | 141.78 | 85.24 | 154.03 | 78.96 | 118.87 | 48.22 |
| **1966** | 31.64 | 112.13 | 151.36 | 136.12 | 178.37 | 236.22 | 165.60 | 155.92 | 156.60 | 111.76 | 63.40 | 5.67 |
| **1967** | 84.08 | 35.82 | 122.16 | 148.75 | 48.04 | 155.43 | 158.43 | 117.80 | 41.47 | 121.03 | 134.89 | 12.90 |
| **1968** | 29.60 | 26.26 | 127.72 | 68.04 | 97.64 | 139.90 | 231.33 | 245.66 | 16.95 | 40.82 | 35.33 | 115.55 |
| **1969** | 68.45 | 93.55 | 137.24 | 149.62 | 99.75 | 356.29 | 197.87 | 234.05 | 102.36 | 51.18 | 69.11 | 29.34 |
| **1970** | 60.82 | 64.93 | 48.78 | 103.26 | 147.29 | 272.72 | 172.00 | 131.77 | 100.73 | 85.52 | 40.23 | 59.55 |
| **1971** | 33.72 | 62.45 | 163.61 | 170.50 | 164.37 | 85.83 | 161.69 | 305.75 | 176.25 | 186.59 | 12.64 | 77.40 |
| **1972** | 49.65 | 105.79 | 178.81 | 194.06 | 162.26 | 205.75 | 349.51 | 113.58 | 277.79 | 37.64 | 73.34 | 66.20 |
| **1973** | 164.22 | 54.22 | 18.38 | 157.67 | 164.53 | 81.95 | 110.00 | 151.44 | 116.85 | 173.57 | 52.35 | 59.21 |
| **1974** | 11.86 | 61.96 | 85.95 | 241.24 | 80.15 | 176.91 | 416.04 | 146.06 | 200.11 | 89.90 | 22.15 | 51.68 |
| **1975** | 78.04 | 87.74 | 127.70 | 156.07 | 120.60 | 117.99 | 203.87 | 181.46 | 123.22 | 258.38 | 120.60 | 68.33 |
| **1976** | 160.00 | 180.17 | 169.57 | 145.19 | 249.76 | 253.65 | 152.97 | 126.47 | 202.78 | 117.64 | 77.72 | 84.08 |
| **1977** | 19.44 | 32.41 | 188.23 | 147.37 | 125.96 | 182.60 | 95.53 | 151.88 | 208.52 | 17.47 | 105.39 | 57.20 |
| **1978** | 24.58 | 45.51 | 66.10 | 154.46 | 120.25 | 186.35 | 123.23 | 90.68 | 191.33 | 126.89 | 76.40 | 30.23 |
| **1979** | 44.80 | 93.61 | 177.19 | 161.81 | 147.10 | 82.24 | 152.79 | 183.21 | 156.13 | 180.54 | 152.45 | 40.12 |
| **1980** | 97.57 | 16.95 | 184.98 | 213.98 | 215.11 | 252.03 | 354.51 | 159.74 | 195.52 | 125.83 | 111.51 | 28.26 |
| **1981** | 17.02 | 55.78 | 216.25 | 263.70 | 126.05 | 122.07 | 240.51 | 261.16 | 169.88 | 245.59 | 127.50 | 14.49 |
| **1982** | 26.17 | 55.84 | 108.44 | 78.03 | 60.33 | 112.43 | 220.61 | 261.25 | 346.75 | 55.34 | 123.64 | 27.17 |
| **1983** | 35.86 | 27.79 | 155.73 | 246.51 | 179.64 | 307.58 | 124.39 | 390.93 | 309.52 | 128.91 | 26.17 | 10.98 |
| **1984** | 27.08 | 67.51 | 71.03 | 87.91 | 74.19 | 261.62 | 36.57 | 149.80 | 60.83 | 23.91 | 54.85 | 32.70 |
| **1985** | 18.15 | 117.77 | 246.35 | 186.50 | 143.25 | 373.00 | 68.73 | 213.92 | 161.79 | 78.00 | 86.11 | 10.43 |
| **1986** | 17.00 | 36.93 | 143.16 | 116.03 | 191.53 | 120.93 | 177.81 | 167.02 | 112.76 | 47.07 | 18.63 | 111.13 |
| **1987** | 56.56 | 33.15 | 147.85 | 47.88 | 167.31 | 97.34 | 134.69 | 182.57 | 184.41 | 50.25 | 31.57 | 18.42 |
| **1988** | 18.58 | 25.09 | 129.16 | 147.13 | 73.41 | 275.36 | 132.57 | 196.38 | 291.78 | 40.89 | 43.05 | 18.58 |
| **1989** | 127.24 | 236.57 | 142.60 | 195.25 | 131.63 | 266.55 | 170.02 | 168.20 | 191.96 | 125.78 | 140.04 | 24.13 |
| **1990** | 48.12 | 141.44 | 103.37 | 99.94 | 211.51 | 180.57 | 122.14 | 134.83 | 287.91 | 66.10 | 100.47 | 15.60 |
| **1991** | 47.77 | 49.55 | 193.21 | 149.72 | 87.69 | 223.51 | 156.14 | 125.48 | 411.38 | 303.72 | 89.12 | 46.70 |
| **1992** | 26.33 | 38.88 | 129.18 | 166.22 | 170.81 | 167.14 | 93.36 | 89.08 | 126.42 | 147.24 | 134.08 | 79.28 |
| **1993** | 68.00 | 101.82 | 62.77 | 59.63 | 94.50 | 190.39 | 395.78 | 104.26 | 184.12 | 177.14 | 127.28 | 90.31 |
| **1994** | 42.77 | 46.63 | 98.73 | 142.15 | 166.59 | 94.87 | 108.06 | 23.80 | 162.41 | 47.92 | 41.81 | 20.26 |
| **1995** | 48.86 | 19.86 | 129.23 | 158.23 | 197.32 | 78.80 | 157.29 | 11.66 | 60.52 | 83.21 | 70.92 | 4.10 |
| **1996** | 43.77 | 25.60 | 254.11 | 39.69 | 66.40 | 162.85 | 245.21 | 108.69 | 95.34 | 93.11 | 103.50 | 105.72 |
| **1997** | 12.61 | 50.44 | 113.86 | 188.40 | 84.19 | 203.61 | 297.07 | 35.23 | 129.81 | 15.95 | 250.34 | 34.49 |
| **1998** | 98.77 | 66.76 | 111.91 | 256.37 | 200.56 | 198.37 | 172.92 | 266.22 | 265.13 | 199.74 | 164 | 21.62 |
| **1999** | 17.79 | 57.58 | 163.04 | 132.96 | 236.47 | 230.01 | 136.84 | 109.02 | 166.28 | 32.67 | 108.69 | 0.65 |
| **2000** | 64.71 | 15.60 | 102.84 | 114.11 | 70.49 | 250.45 | 118.44 | 112.37 | 204.52 | 131.15 | 135.19 | 12.13 |
| **2001** | 129.84 | 66.67 | 71.59 | 43.51 | 83.52 | 140.72 | 19.30 | 184.58 | 307.06 | 221.43 | 104.57 | 37.20 |
| **2002** | 75.32 | 25.11 | 114.46 | 43.19 | 87.19 | 151.71 | 215.15 | 82.87 | 133.09 | 156.03 | 13.50 | 53.18 |
| **2003** | 90.71 | 37.59 | 133.89 | 149.42 | 155.95 | 78.59 | 437.70 | 518.47 | 134.82 | 86.67 | 208.44 | 24.54 |
| **2004** | 14.01 | 48.53 | 76.56 | 119.38 | 134.18 | 332.72 | 90.32 | 121.20 | 150.01 | 496.74 | 95.51 | 81.23 |
