# Supplementary material for: Qualitative simulation of bathymetric changes due to reservoir sedimentation: A Japanese case study
Source: PLoS One. 2017 Apr 6;12(4):e0174931. doi: 10.1371/journal.pone.0174931 (PMC5383045; doi:10.1371/journal.pone.0174931)
Supplement: S3 Table — (DOCX) [file pone.0174931.s003.docx]

# HEC – RAS geometry data (x-coordinates, elevations and lengths)

Table S3: Geometric data of Tenryu River

| **River St.** | **X-coord.** | **Elevation** | **LoB** | **Channel** | **RoB** | **River St.** | **X-coord.** | **Elevation** | **LoB** | **Channel** | **RoB** |
| --- | --- | --- | --- | --- | --- | --- | --- | --- | --- | --- | --- |
| **100** | 5988 | 280.17 | 450 | 450 | 450 | **91** | 4399 | 279.18 | 477 | 507 | 540 |
|  | 5994 | 260 |  |  |  |  | 4409 | 253 |  |  |  |
|  | 6090 | 260 |  |  |  |  | 4480 | 253 |  |  |  |
|  | 6098 | 280.17 |  |  |  |  | 4489 | 279.18 |  |  |  |
| **99** | 5881 | 280.06 | 314 | 352 | 395 | **90** | 4199 | 279.07 | 350 | 301 | 253 |
|  | 5888 | 259.3 |  |  |  |  | 4210 | 252.2 |  |  |  |
|  | 5963 | 259.3 |  |  |  |  | 433  0 | 252.2 |  |  |  |
|  | 5971 | 280.06 |  |  |  |  | 4339 | 279.07 |  |  |  |
| **98** | 5568 | 279.95 | 353 | 378 | 404 | **89** | 4350 | 278.96 | 897 | 906 | 915 |
|  | 5575 | 259 |  |  |  |  | 4358 | 251.4 |  |  |  |
|  | 5690 | 259 |  |  |  |  | 4417 | 251.4 |  |  |  |
|  | 5698 | 279.95 |  |  |  |  | 4426 | 278.96 |  |  |  |
| **97** | 5221 | 279.84 | 422 | 379 | 310 | **88** | 4506 | 278.85 | 295 | 405 | 428 |
|  | 5228 | 258.5 |  |  |  |  | 4513 | 250.3 |  |  |  |
|  | 5320 | 258.5 |  |  |  |  | 4558 | 250.3 |  |  |  |
|  | 5328 | 279.84 |  |  |  |  | 4567 | 278.85 |  |  |  |
| **96** | 4873 | 279.73 | 1158 | 1162 | 1164 | **87** | 4266 | 278.74 | 1360 | 1334 | 1309 |
|  | 4879 | 258 |  |  |  |  | 4275 | 249 |  |  |  |
|  | 4990 | 258 |  |  |  |  | 4334 | 249 |  |  |  |
|  | 4999 | 279.73 |  |  |  |  | 4343 | 278.74 |  |  |  |
| **95** | 3946 | 279.62 | 349 | 306 | 263 | **86** | 3224 | 278.63 | 922 | 890 | 859 |
|  | 3956 | 257 |  |  |  |  | 3230 | 247 |  |  |  |
|  | 4026 | 257 |  |  |  |  | 3303 | 247 |  |  |  |
|  | 4034 | 279.62 |  |  |  |  | 3311 | 278.63 |  |  |  |
| **94** | 3776 | 279.51 | 342 | 321 | 301 | **85** | 3982 | 278.62 | 426 | 522 | 535 |
|  | 3782 | 256.3 |  |  |  |  | 3988 | 244 |  |  |  |
|  | 3847 | 256.3 |  |  |  |  | 4057 | 244 |  |  |  |
|  | 3853 | 279.51 |  |  |  |  | 4062 | 278.62 |  |  |  |
| **93** | 3996 | 279.4 | 696 | 780 | 823 | **84** | 4078 | 278.41 | 981 | 1016 | 1052 |
|  | 4005 | 255.8 |  |  |  |  | 4085 | 242 |  |  |  |
|  | 4106 | 255.8 |  |  |  |  | 4175 | 242 |  |  |  |
|  | 4112 | 279.4 |  |  |  |  | 4183 | 278.41 |  |  |  |
| **92** | 3938 | 279.29 | 580 | 560 | 541 | **83** | 4791 | 278.3 | 826 | 852 | 878 |
|  | 3946 | 254.6 |  |  |  |  | 4798 | 238.3 |  |  |  |
|  | 4030 | 254.6 |  |  |  |  | 4930 | 238.3 |  |  |  |
|  | 4037 | 279.29 |  |  |  |  | 4937 | 278.3 |  |  |  |

Continued..

| **River St.** | **X-coord.** | **Elevation** | **LoB** | **Channel** | **RoB** | **River St.** | **X-coord.** | **Elevation** | **LoB** | **Channel** | **RoB** |
| --- | --- | --- | --- | --- | --- | --- | --- | --- | --- | --- | --- |
| **82** | 3965 | 278.19 | 465 | 393 | 330 | **73** | 2882 | 277.2 | 816 | 880 | 944 |
|  | 3973 | 237.6 |  |  |  |  | 2890 | 201 |  |  |  |
|  | 4080 | 237.6 |  |  |  |  | 3048 | 201 |  |  |  |
|  | 4090 | 278.19 |  |  |  |  | 3056 | 277.2 |  |  |  |
| **81** | 3844 | 278.08 | 464 | 441 | 428 | **72** | 3446 | 277.09 | 1170 | 1185 | 1201 |
|  | 3852 | 237 |  |  |  |  | 3457 | 193 |  |  |  |
|  | 4065 | 237 |  |  |  |  | 3600 | 193 |  |  |  |
|  | 4073 | 278.08 |  |  |  |  | 3612 | 277.09 |  |  |  |
| **80** | 4196 | 277.97 | 1207 | 1220 | 1234 | **71** | 2987 | 276.98 | 901 | 872 | 844 |
|  | 4204 | 236.4 |  |  |  |  | 2994 | 188.5 |  |  |  |
|  | 4290 | 236.4 |  |  |  |  | 3200 | 188.5 |  |  |  |
|  | 4301 | 277.97 |  |  |  |  | 3209 | 276.98 |  |  |  |
| **79** | 3087 | 277.86 | 1111 | 1088 | 1065 | **70** | 3672 | 276.87 | 1029 | 1135 | 1243 |
|  | 3097 | 233 |  |  |  |  | 3680 | 180 |  |  |  |
|  | 3210 | 233 |  |  |  |  | 3800 | 180 |  |  |  |
|  | 3219 | 277.86 |  |  |  |  | 3814 | 276.87 |  |  |  |
| **78** | 2692 | 277.75 | 556 | 607 | 659 | **69** | 2764 | 276.76 | 773 | 738 | 703 |
|  | 2699 | 228.3 |  |  |  |  | 2772 | 178 |  |  |  |
|  | 2810 | 228.3 |  |  |  |  | 2958 | 178 |  |  |  |
|  | 2821 | 277.75 |  |  |  |  | 2968 | 276.76 |  |  |  |
| **77** | 2617 | 277.64 | 960 | 948 | 936 | **68** | 2159 | 276.65 | 819 | 682 | 547 |
|  | 2629 | 220 |  |  |  |  | 2169 | 173 |  |  |  |
|  | 2806 | 220 |  |  |  |  | 2397 | 173 |  |  |  |
|  | 2817 | 277.64 |  |  |  |  | 2407 | 276.65 |  |  |  |
| **76** | 1661 | 277.53 | 1107 | 998 | 890 | **67** | 1880 | 276.54 | 680 | 707 | 734 |
|  | 1669 | 214 |  |  |  |  | 1889 | 167 |  |  |  |
|  | 1910 | 214 |  |  |  |  | 2075 | 167 |  |  |  |
|  | 1921 | 277.53 |  |  |  |  | 2085 | 276.54 |  |  |  |
| **75** | 1430 | 277.42 | 944 | 874 | 805 | **66** | 2117 | 276.43 | 1306 | 1443 | 1580 |
|  | 1439 | 209 |  |  |  |  | 2129 | 163 |  |  |  |
|  | 1560 | 209 |  |  |  |  | 2334 | 163 |  |  |  |
|  | 1569 | 277.42 |  |  |  |  | 2344 | 276.43 |  |  |  |
| **74** | 2139 | 277.31 | 751 | 763 | 775 | **65** | 1285 | 276.32 | 1186 | 1174 | 1163 |
|  | 2149 | 206 |  |  |  |  | 1287 | 158 |  |  |  |
|  | 2290 | 206 |  |  |  |  | 1531 | 158 |  |  |  |
|  | 2300 | 277.31 |  |  |  |  | 1533 | 276.32 |  |  |  |

| **River St.** | **X-coord.** | **Elevation** | **LoB** | **Channel** | **RoB** | **River St.** | **X-coord.** | **Elevation** | **LoB** | **Channel** | **RoB** |
| --- | --- | --- | --- | --- | --- | --- | --- | --- | --- | --- | --- |
| **64** | 396 | 276.21 | 1094 | 928 | 764 | **56** | 362 | 275.33 | 243 | 164 | 86 |
|  | 408 | 152 |  |  |  |  | 373 | 128 |  |  |  |
|  | 625 | 152 |  |  |  |  | 719 | 128 |  |  |  |
|  | 633 | 276.21 |  |  |  |  | 728 | 275.33 |  |  |  |
| **63** | 100 | 276.1 | 721 | 710 | 700 | **55** | 319 | 275.22 | 231 | 200 | 224 |
|  | 110 | 150 |  |  |  |  | 328 | 125.3 |  |  |  |
|  | 334 | 150 |  |  |  |  | 621 | 125.3 |  |  |  |
|  | 342 | 276.1 |  |  |  |  | 632 | 275.22 |  |  |  |
| **62** | 692 | 275.99 | 562 | 638 | 719 | **54** | 168 | 275.11 | 159 | 90 | 149 |
|  | 699 | 146 |  |  |  |  | 177 | 122.6 |  |  |  |
|  | 944 | 146 |  |  |  |  | 690 | 122.6 |  |  |  |
|  | 952 | 275.99 |  |  |  |  | 699 | 275.11 |  |  |  |
| **61** | 596 | 275.88 | 1458 | 1447 | 1437 | **53** | 267 | 275 | 0 | 0 | 0 |
|  | 607 | 141 |  |  |  |  | 276 | 120 |  |  |  |
|  | 930 | 141 |  |  |  |  | 540 | 120 |  |  |  |
|  | 939 | 275.88 |  |  |  |  | 550 | 275 |  |  |  |
| **60** | 951 | 275.77 | 107 | 125 | 185 |  |  |  |  |  |  |
|  | 959 | 139 |  |  |  |  |  |  |  |  |  |
|  | 1207 | 139 |  |  |  |  |  |  |  |  |  |
|  | 1217 | 275.77 |  |  |  |  |  |  |  |  |  |
| **59** | 852 | 275.66 | 120 | 190 | 263 |  |  |  |  |  |  |
|  | 861 | 136 |  |  |  |  |  |  |  |  |  |
|  | 1240 | 136 |  |  |  |  |  |  |  |  |  |
|  | 1250 | 275.66 |  |  |  |  |  |  |  |  |  |
| **58** | 742 | 275.55 | 223 | 232 | 241 |  |  |  |  |  |  |
|  | 750 | 133.3 |  |  |  |  |  |  |  |  |  |
|  | 1117 | 133.3 |  |  |  |  |  |  |  |  |  |
|  | 1127 | 275.55 |  |  |  |  |  |  |  |  |  |
| **57** | 554 | 275.44 | 237 | 195 | 155 |  |  |  |  |  |  |
|  | 561 | 130.6 |  |  |  |  |  |  |  |  |  |
|  | 924 | 130.6 |  |  |  |  |  |  |  |  |  |
|  | 932 | 275.44 |  |  |  |  |  |  |  |  |  |
