# Supplementary material for: Qualitative simulation of bathymetric changes due to reservoir sedimentation: A Japanese case study
Source: PLoS One. 2017 Apr 6;12(4):e0174931. doi: 10.1371/journal.pone.0174931 (PMC5383045; doi:10.1371/journal.pone.0174931)
Supplement: S4 Table — (DOCX) [file pone.0174931.s004.docx]

# Precipitation data in the catchment of Tenryu River

Table S4: Monthly Precipitation data (mm) [1].

| **Year** | **Jan.** | **Feb.** | **Mar.** | **Apr.** | **May** | **Jun.** | **Jul.** | **Aug.** | **Sep.** | **Oct.** | **Nov.** | **Dec.** |
| --- | --- | --- | --- | --- | --- | --- | --- | --- | --- | --- | --- | --- |
| **1957** | 46.7 | 111.3 | 60.7 | 174.9 | 273.9 | 370.4 | 250.9 | 394.8 | 409.7 | 88.2 | 144.7 | 108.9 |
| **1958** | 120.6 | 145.7 | 99.8 | 152.4 | 116.2 | 60.4 | 352.3 | 217.5 | 382.2 | 226 | 95.4 | 137 |
| **1959** | 137.5 | 272.6 | 143.8 | 387.5 | 186.8 | 132.3 | 201.6 | 576.9 | 277.2 | 161.6 | 147.5 | 183.4 |
| **1960** | 37.1 | 18.1 | 150.8 | 249 | 234.3 | 217.7 | 264.2 | 371.8 | 166.6 | 112.7 | 144.7 | 63.1 |
| **1961** | 87.8 | 38.4 | 201.2 | 397.8 | 297.1 | 512.2 | 81.2 | 197.2 | 97.5 | 230.4 | 162.8 | 49.7 |
| **1962** | 29.2 | 16.9 | 81.9 | 307.7 | 329.3 | 422 | 341.5 | 313.6 | 146.8 | 159.3 | 214.4 | 124.1 |
| **1963** | 5.3 | 36.3 | 116.7 | 235.6 | 458.2 | 334.2 | 206.7 | 200.2 | 129.7 | 181.5 | 57.2 | 39.2 |
| **1964** | 177.6 | 93.5 | 124.5 | 179.1 | 96.2 | 509.9 | 49.3 | 101.5 | 319.7 | 106.6 | 36.3 | 48.9 |
| **1965** | 43.2 | 37.3 | 49.7 | 174.2 | 431.1 | 251.2 | 230.2 | 138.4 | 250.1 | 128.2 | 193 | 78.3 |
| **1966** | 51.3 | 181.8 | 245.4 | 220.7 | 289.2 | 383 | 268.5 | 252.8 | 253.9 | 181.2 | 102.8 | 9.2 |
| **1967** | 148.6 | 63.3 | 215.9 | 262.9 | 84.9 | 274.7 | 280 | 208.2 | 73.3 | 213.9 | 238.4 | 22.8 |
| **1968** | 62 | 55 | 267.5 | 142.5 | 204.5 | 293 | 484.5 | 514.5 | 35.5 | 85.5 | 74 | 242 |
| **1969** | 105 | 143.5 | 210.5 | 229.5 | 153 | 546.5 | 303.5 | 359 | 157 | 78.5 | 106 | 45 |
| **1970** | 96 | 102.5 | 77 | 163 | 232.5 | 430.5 | 271.5 | 208 | 159 | 135 | 63.5 | 94 |
| **1971** | 44 | 81.5 | 213.5 | 222.5 | 214.5 | 112 | 211 | 399 | 230 | 243.5 | 16.5 | 101 |
| **1972** | 76.5 | 163 | 275.5 | 299 | 250 | 317 | 538.5 | 175 | 428 | 58 | 113 | 102 |
| **1973** | 263.5 | 87 | 29.5 | 253 | 264 | 131.5 | 176.5 | 243 | 187.5 | 278.5 | 84 | 0 |
| **1974** | 22.5 | 117.5 | 163 | 457.5 | 152 | 335.5 | 789 | 277 | 379.5 | 170.5 | 42 | 98 |
| **1975** | 104.5 | 117.5 | 171 | 209 | 161.5 | 158 | 273 | 243 | 165 | 346 | 161.5 | 91.5 |
| **1976** | 0 | 255 | 240 | 205.5 | 353.5 | 359 | 216.5 | 179 | 287 | 166.5 | 110 | 119 |
| **1977** | 34.5 | 57.5 | 334 | 261.5 | 223.5 | 324 | 169.5 | 269.5 | 370 | 31 | 187 | 101.5 |
| **1978** | 37 | 68.5 | 99.5 | 232.5 | 181 | 280.5 | 185.5 | 136.5 | 288 | 191 | 115 | 45.5 |
| **1979** | 67 | 140 | 265 | 242 | 220 | 123 | 228.5 | 274 | 233.5 | 270 | 228 | 60 |
| **1980** | 129.5 | 22.5 | 245.5 | 284 | 285.5 | 334.5 | 470.5 | 212 | 259.5 | 167 | 148 | 37.5 |
| **1981** | 23.5 | 77 | 298.5 | 364 | 174 | 168.5 | 332 | 360.5 | 234.5 | 339 | 176 | 20 |
| **1982** | 52.5 | 112 | 217.5 | 156.5 | 121 | 225.5 | 442.5 | 524 | 695.5 | 111 | 248 | 54.5 |
| **1983** | 55.5 | 43 | 241 | 381.5 | 278 | 476 | 192.5 | 605 | 479 | 199.5 | 40.5 | 17 |
| **1984** | 38.5 | 96 | 101 | 125 | 105.5 | 372 | 52 | 213 | 86.5 | 34 | 78 | 46.5 |
| **1985** | 23.5 | 152.5 | 319 | 241.5 | 185.5 | 483 | 89 | 277 | 209.5 | 101 | 111.5 | 13.5 |
| **1986** | 26 | 56.5 | 219 | 177.5 | 293 | 185 | 272 | 255.5 | 172.5 | 72 | 28.5 | 170 |
| **1987** | 107.5 | 63 | 281 | 91 | 318 | 185 | 256 | 347 | 350.5 | 95.5 | 60 | 35 |
| **1988** | 30 | 40.5 | 208.5 | 237.5 | 118.5 | 444.5 | 214 | 317 | 471 | 66 | 69.5 | 1 |
| **1989** | 174 | 323.5 | 195 | 267 | 180 | 364.5 | 232.5 | 230 | 262.5 | 172 | 191.5 | 33 |
| **1990** | 91 | 267.5 | 195.5 | 189 | 400 | 341.5 | 231 | 255 | 544.5 | 125 | 190 | 29.5 |
| **1991** | 67 | 69.5 | 271 | 210 | 123 | 313.5 | 219 | 176 | 577 | 426 | 125 | 65.5 |
| **1992** | 43 | 63.5 | 211 | 271.5 | 279 | 273 | 152.5 | 145.5 | 206.5 | 240.5 | 219 | 129.5 |
| **1993** | 97.5 | 146 | 90 | 85.5 | 135.5 | 273 | 567.5 | 149.5 | 264 | 254 | 182.5 | 129.5 |
| **1994** | 66.5 | 72.5 | 153.5 | 221 | 259 | 147.5 | 168 | 37 | 252.5 | 74.5 | 65 | 31.5 |
| **1995** | 77.5 | 31.5 | 205 | 251 | 313 | 125 | 249.5 | 18.5 | 96 | 132 | 112.5 | 6.5 |
| **1996** | 59 | 34.5 | 342.5 | 53.5 | 89.5 | 219.5 | 330.5 | 146.5 | 128.5 | 125.5 | 139.5 | 142.5 |
| **1997** | 17 | 68 | 153.5 | 254 | 113.5 | 274.5 | 400.5 | 47.5 | 175 | 21.5 | 337.5 | 46.5 |
| **1998** | 180.5 | 122 | 204.5 | 468.5 | 366.5 | 362.5 | 316 | 486.5 | 484.5 | 365 | 3 | 39.5 |
| **1999** | 27.5 | 89 | 252 | 205.5 | 365.5 | 355.5 | 211.5 | 168.5 | 257 | 50.5 | 168 | 1 |
| **2000** | 112 | 27 | 178 | 197.5 | 122 | 433.5 | 205 | 194.5 | 354 | 227 | 234 | 21 |
| **2001** | 185 | 95 | 102 | 62 | 119 | 200.5 | 27.5 | 263 | 437.5 | 315.5 | 149 | 53 |
| **2002** | 139.5 | 46.5 | 212 | 80 | 161.5 | 281 | 398.5 | 153.5 | 246.5 | 289 | 25 | 98.5 |
| **2003** | 146 | 60.5 | 215.5 | 240.5 | 251 | 126.5 | 704.5 | 834.5 | 217 | 139.5 | 335.5 | 39.5 |
| **2004** | 27 | 93.5 | 147.5 | 230 | 258.5 | 641 | 174 | 233.5 | 289 | 957 | 184 | 156.5 |

# References

| [1] | JMA, "Japan Mateorological Agency," 2012. [Online]. Available: http://www.data.jma.go.jp/obd/stats/etrn/view/monthly_s3_en.php?block_no=47656&view=13. [Accessed 04 May 2012]. |
| --- | --- |
