# Supplementary material for: Qualitative simulation of bathymetric changes due to reservoir sedimentation: A Japanese case study
Source: PLoS One. 2017 Apr 6;12(4):e0174931. doi: 10.1371/journal.pone.0174931 (PMC5383045; doi:10.1371/journal.pone.0174931)
Supplement: S5 Table — (DOCX) [file pone.0174931.s005.docx]

# Temperature in the region around Sakuma (⁰C)

Table S5: Average monthly temperature in the region around Sakuma Dam (Celsius) [1]

| **Year** | **Jan.** | **Feb.** | **Mar.** | **Apr.** | **May** | **Jun.** | **Jul.** | **Aug.** | **Sep.** | **Oct.** | **Nov.** | **Dec.** |
| --- | --- | --- | --- | --- | --- | --- | --- | --- | --- | --- | --- | --- |
| **1957** | 7.8 | 5.6 | 8.3 | 14.5 | 17 | 20.3 | 24.7 | 26.6 | 21.1 | 17.9 | 13.7 | 9.1 |
| **1958** | 6.1 | 7.6 | 9.8 | 15.2 | 18 | 22 | 25 | 26.3 | 23.8 | 16.9 | 12.7 | 9.4 |
| **1959** | 5.5 | 9.2 | 10.7 | 15.1 | 18.5 | 20.8 | 26 | 26.4 | 24.5 | 18.5 | 14.2 | 9 |
| **1960** | 6.2 | 8 | 10.8 | 14 | 18.2 | 21.5 | 25.8 | 26.6 | 24 | 18.6 | 14.1 | 7.8 |
| **1961** | 5.1 | 5.7 | 9.5 | 15.1 | 18.9 | 22.2 | 27 | 26.8 | 25.2 | 20.4 | 14.3 | 8.5 |
| **1962** | 5.5 | 6.8 | 9.3 | 14 | 17.7 | 21.2 | 25.2 | 27.3 | 24.2 | 17.5 | 12.7 | 8.9 |
| **1963** | 3.3 | 4.8 | 8.6 | 14.5 | 18.6 | 22.4 | 25.6 | 26.1 | 21.5 | 17.6 | 13.3 | 9.2 |
| **1964** | 7 | 5.8 | 8.7 | 16.4 | 19.1 | 21.2 | 25.6 | 27.1 | 23.5 | 17.9 | 12.7 | 8.4 |
| **1965** | 6.4 | 6.2 | 7.9 | 11.6 | 18 | 22.3 | 25.6 | 26.3 | 22.1 | 17.2 | 14.3 | 7.8 |
| **1966** | 5.3 | 8.7 | 10.8 | 14.7 | 17.7 | 20.6 | 25 | 26.4 | 23.7 | 18.3 | 13.5 | 7 |
| **1967** | 5.1 | 5.5 | 9.8 | 14.3 | 19.1 | 22.6 | 25.8 | 27.1 | 23.7 | 17.6 | 14.2 | 6.8 |
| **1968** | 6 | 4.2 | 10.2 | 14.1 | 17.7 | 21 | 24.2 | 26.2 | 22.6 | 17.5 | 13.7 | 10.5 |
| **1969** | 6.8 | 7.5 | 9.1 | 14.9 | 19.6 | 20.9 | 24.3 | 26.6 | 23.4 | 18 | 13.2 | 7.7 |
| **1970** | 5.4 | 7.4 | 6.9 | 13.5 | 19 | 20.7 | 25.1 | 26.3 | 24.4 | 18.3 | 13.3 | 7.9 |
| **1971** | 6.3 | 7.4 | 9.3 | 14 | 18.3 | 21.7 | 25.9 | 27 | 23.1 | 17.2 | 13.2 | 9.3 |
| **1972** | 9 | 8 | 10.3 | 14.3 | 19 | 22.1 | 25.8 | 26.7 | 23.6 | 19.4 | 13.6 | 9.1 |
| **1973** | 7.7 | 8.2 | 9.5 | 16.7 | 18.1 | 20.7 | 25.8 | 27.3 | 22.7 | 17.9 | 12.5 | 6.6 |
| **1974** | 4.9 | 7.1 | 8.9 | 15.3 | 19.1 | 22.4 | 24.1 | 27.1 | 22.9 | 18.7 | 12.6 | 8.2 |
| **1975** | 5.9 | 6.1 | 9.3 | 14.7 | 18.7 | 22.1 | 25.5 | 26.3 | 25.1 | 18.3 | 14 | 7.6 |
| **1976** | 5.6 | 8.6 | 10.3 | 13.7 | 18.2 | 21.9 | 24.2 | 26.2 | 22.6 | 18.1 | 12.7 | 8.1 |
| **1977** | 4.6 | 5.7 | 10.8 | 15.4 | 18.7 | 21.8 | 25.8 | 25.7 | 24.4 | 19.4 | 15.4 | 9.8 |
| **1978** | 6.8 | 5.5 | 9.2 | 14.4 | 18.8 | 22.9 | 27.1 | 27.8 | 23.8 | 18.5 | 13.7 | 9.1 |
| **1979** | 7.3 | 9.4 | 10.2 | 14.5 | 17.9 | 23.7 | 24.8 | 27 | 24.3 | 19.6 | 14.9 | 10.1 |
| **1980** | 6.5 | 5.7 | 9.7 | 14.3 | 18.7 | 22.9 | 24.8 | 24.9 | 23 | 18.6 | 14.2 | 7.3 |
| **1981** | 4.4 | 6.3 | 10.1 | 14.4 | 18.1 | 21.7 | 25.9 | 26.1 | 22.1 | 17.7 | 11.7 | 8 |
| **1982** | 6.3 | 6.4 | 10.5 | 14.3 | 20 | 21.3 | 22.7 | 25.6 | 22.5 | 18 | 15 | 9.3 |
| **1983** | 6.5 | 6.2 | 9.3 | 15.7 | 19.4 | 21.1 | 24.9 | 27.2 | 23.6 | 18.3 | 12.2 | 6.9 |
| **1984** | 4.4 | 4.3 | 6.7 | 13.1 | 17.7 | 22 | 26.4 | 27.4 | 23.6 | 18.3 | 13.5 | 8.5 |
| **1985** | 4.8 | 7.4 | 10.6 | 15 | 19.1 | 20.8 | 26.9 | 26.9 | 23.8 | 18.4 | 13.1 | 7.8 |
| **1986** | 4.6 | 5.1 | 9.2 | 14.3 | 17.8 | 21.3 | 24.6 | 26.3 | 24.1 | 17.5 | 13.5 | 9.5 |
| **1987** | 7 | 7.6 | 10.6 | 14.6 | 18.8 | 21.9 | 26.4 | 27 | 23.7 | 19.6 | 14.2 | 9.3 |
| **1988** | 8.3 | 6.3 | 9.7 | 14.6 | 18.3 | 22.1 | 23.5 | 26.4 | 24 | 18 | 11.7 | 7.7 |
| **1989** | 8.9 | 8.4 | 10.2 | 15.1 | 17.9 | 20.7 | 24.4 | 26.9 | 24.9 | 18.3 | 13.8 | 9 |
| **1990** | 6.5 | 9.5 | 10.9 | 15 | 18.2 | 22.8 | 25.8 | 27.8 | 24.7 | 19.6 | 15.1 | 9.4 |
| **1991** | 6.4 | 6.3 | 10.7 | 15.6 | 18.5 | 23.5 | 26.6 | 26.2 | 24.6 | 19.2 | 13.8 | 9.7 |
| **1992** | 7.9 | 7.7 | 11.3 | 15.5 | 17.3 | 21.1 | 25.3 | 26.2 | 23.5 | 17.9 | 13.9 | 9.5 |
| **1993** | 7.9 | 8.8 | 9.4 | 14 | 17.7 | 21.5 | 23.2 | 25.5 | 22.9 | 17.7 | 14.4 | 9.5 |
| **1994** | 7.3 | 6.8 | 9.2 | 15.9 | 19.4 | 22.2 | 27.9 | 28.1 | 24.7 | 20.7 | 14.4 | 10.1 |
| **1995** | 6.7 | 7.1 | 10 | 14.7 | 18.6 | 21.2 | 26.2 | 28.7 | 23.6 | 19.8 | 12.5 | 7.3 |
| **1996** | 6.8 | 5.8 | 9.9 | 12.2 | 18.3 | 22.4 | 25.8 | 26.3 | 22.9 | 18.3 | 14.6 | 8.7 |
| **1997** | 6.4 | 7.2 | 11.3 | 15.1 | 18.8 | 21.8 | 26 | 26.8 | 24 | 18.2 | 14.8 | 9.9 |
| **1998** | 6.4 | 8.4 | 11 | 17.1 | 20.9 | 22.6 | 25.8 | 27.5 | 24.6 | 20.6 | 14.4 | 9.9 |
| **1999** | 6.8 | 7 | 11.2 | 14.8 | 19.3 | 22.2 | 25.8 | 27.6 | 25.9 | 20.3 | 14.5 | 8.9 |
| **2000** | 8.3 | 5.9 | 9.6 | 14.9 | 19.6 | 21.8 | 26.2 | 27.2 | 24.4 | 19.3 | 14.9 | 9 |
| **2001** | 5.9 | 7.3 | 10.4 | 15.3 | 19.1 | 23.3 | 27.5 | 26.6 | 23.6 | 19.2 | 12.8 | 8.2 |
| **2002** | 7.4 | 7.8 | 12.3 | 16.4 | 18.7 | 21.4 | 27.3 | 27.9 | 23.5 | 18.8 | 11.9 | 8.8 |
| **2003** | 6.1 | 7.7 | 9.5 | 16 | 18.8 | 22.8 | 23.4 | 25.9 | 24.7 | 18 | 15.8 | 9.5 |
| **2004** | 6.2 | 8.4 | 10.4 | 16.3 | 20.3 | 23.6 | 27.8 | 27 | 25.4 | 18.7 | 15.6 | 10.8 |

# References

| [1] | JMA, "Japan Mateorological Agency," 2012. [Online]. Available: http://www.data.jma.go.jp/obd/stats/etrn/view/monthly_s3_en.php?block_no=47656&view=13. [Accessed 04 May 2012]. |
| --- | --- |
